# Early Differential Diagnosis of Rhino-Orbito-Cerebral Mucormycosis and Bacterial Orbital Cellulitis: Based on Computed Tomography Findings

**DOI:** 10.1371/journal.pone.0160897

**Published:** 2016-08-08

**Authors:** Jun Hyuk Son, Hyung Bin Lim, Soo Hyun Lee, Jae Wook Yang, Sung Bok Lee

**Affiliations:** 1 Department of Ophthalmology, Yeungnam University College of Medicine, Daegu, Republic of Korea; 2 Department of Ophthalmology, Chungnam National University College of Medicine, Daejeon, Republic of Korea; 3 Department of Ophthalmology, Armed Forces Capital Hospital, Seongnam, Republic of Korea; 4 Department of Ophthalmology, Busan Paik Hospital, Inje University College of Medicine, Busan, Republic of Korea; 5 Research Institute for Medical Science, Chungnam National University College of Medicine, Daejeon, Republic of Korea; University of Birmingham, UNITED KINGDOM

## Abstract

**Purpose:**

To identify significant clinical and radiological findings that distinguish rhino-orbito-cerebral mucormycosis (ROCM) from bacterial orbital cellulitis (BOC).

**Methods:**

This study was retrospective, multicenter, case-control study that enrolled 34 cases; 14 cases were diagnosed with ROCM and 20 cases were diagnosed with BOC at three different tertiary hospitals between 2005 and 2013. The medical records of all 34 cases were reviewed. The initial clinical manifestations (eyelid swelling, ptosis, extraocular muscle [EOM] limitation, conjunctival injection, and chemosis) and computed tomography (CT) findings (sinus mucosal thickening, full opacification, and air-fluid level) of both diseases were compared.

**Results:**

Patients with diabetes mellitus (DM) and hypertension (HTN) showed higher incidence rates of ROCM than BOC (DM: *p* < 0.001, HTN: *p* = 0.036). ROCM cases exhibited more frequent EOM limitation than cases with BOC (100.0% vs. 66.7%, *p* = 0.024) but less frequent eyelid swelling (35.7% vs. 90.0%, *p* = 0.002). However, the incidence rates of ptosis, conjunctival injection, and chemosis exhibited no differences between the diseases. Abnormal CT findings were observed in the sinuses of all patients with ROCM, whereas 12 patients with BOC had sinus abnormalities (100.0% vs. 60.0%, *p* = 0.011). Thickening of the sinus mucosa was more frequent in patients with ROCM than in those with BOC (92.9% vs. 45.0%, *p* = 0.009). No significant differences in full opacification or air-fluid level were detected between the groups.

**Conclusions:**

The differential diagnosis of ROCM and BOC is difficult. Nevertheless, physicians should consider ROCM when a patient with suspected orbital cellulitis presents with EOM limitation without swollen eyelids or thickening of the sinus mucosa on a CT scan.

## Introduction

Mucormycosis is an infection caused by members of the order Mucorales [1] and is primarily found in patients with poorly controlled diabetes, particularly those with ketoacidosis, and in immunocompromised patients, such as those with hematological malignancies or recipients of hematopoietic stem cell transplants or solid organ transplants.[2] Mucormycosis is relatively uncommon but can result in acute disease that rapidly advances into adjacent tissues and organs, resulting in death unless aggressive treatment with antifungal agents and surgical excision is instituted during the early phase of the infection.[3–5]

Rhino-orbito-cerebral mucormycosis (ROCM) is a subtype of mucormycosis. This infection develops after fungal sporangiospores are inhaled into the paranasal sinus. The infection can spread to the orbits and cavernous sinuses, thereby extending into the cranial cavity. Early signs and symptoms of ROCM are similar to those of sinusitis and periorbital cellulitis. Patients with a progressing infection have cranial nerve palsy, eyelid swelling, ptosis, conjunctival injection, chemosis, proptosis, limited eye movement, and decreased vision. Symptoms of brain involvement include headache, confusion, hemiparesis, and seizure. The mortality rate due to ROCM is very high due to its rapid spread.[6]

Most cases of ROCM develop from ethmoid sinusitis, where the disease may spread rapidly through the structurally defective lamina papyracea into the orbit. The ocular symptoms of ROCM, such as facial edema, pain, and blepharoptosis, are similar to those of bacterial orbital cellulitis (BOC) soon after infection onset. As BOC occurs more frequently than ROCM but its early symptoms are similar, it is difficult for clinicians to identify ROCM in a patient with these ocular symptoms. Mucormycosis is characterized by vascular invasion and “black eschar”, which is a progressive formation of necrotic tissue, but these clinical features are manifested during disease progression; thus, ROCM and BOC cannot be distinguished easily at onset.[7]

An early diagnosis is crucial, as the treatment modalities for these diseases are quite different, and a delayed ROCM diagnosis can result in fatal consequences. We wanted to identify useful clinical signs to help with an early diagnosis by comparing the diverse clinical manifestations and radiological findings in patients diagnosed with ROCM and BOC during their first hospital visit.

## Materials and Methods

### Subjects

Thirty-four patients, who were diagnosed with ROCM and BOC between 2005 and 2013 at three tertiary medical institutions in South Korea (Chungnam National University Hospital, Inje University Busan Paik Hospital, and Yeungnam University Medical Center), were included in this retrospective medical record review. The study protocol was approved by the Institutional Review Board of each hospital (Chungnam National University Hospital, Inje University Busan Paik Hospital, and Yeungnam University Medical Center). The study adhered to the tenets of the Declaration of Helsinki. ROCM was confirmed by histopathologic examination using Grocott's methenamine silver, periodic acid—Schiff, Periodic acid—Schiff—diastase staining, and cases without a histopathological diagnosis were excluded. If there is a possibility of other fungal infections, it was also excluded. Cases of BOC that involved behind the septum were included in this study, whereas those with preseptal cellulitis were excluded. BOC was diagnosed based on clinical manifestations and computed tomography (CT) findings, and all patients with BOC recovered completely after antibiotic therapy.

We reviewed the medical records of all patients to compare the medical history and the clinical manifestations of ROCM and BOC, including eyelid swelling, ptosis without swelling, extraocular muscle (EOM) limitation, conjunctival injection, and chemosis. The following patients were excluded from the study: those undergoing ophthalmic or nasal surgery and those with a traumatic infection, other infections associated with surgery for ocular adnexa (e.g., intraocular, periocular, and eyelids), and dental treatment. Patients with BOC and complications, such as thrombophlebitis, subperiosteal abscess, or an orbital abscess were also excluded from the study.

### Radiological analysis

**Fig 1:**
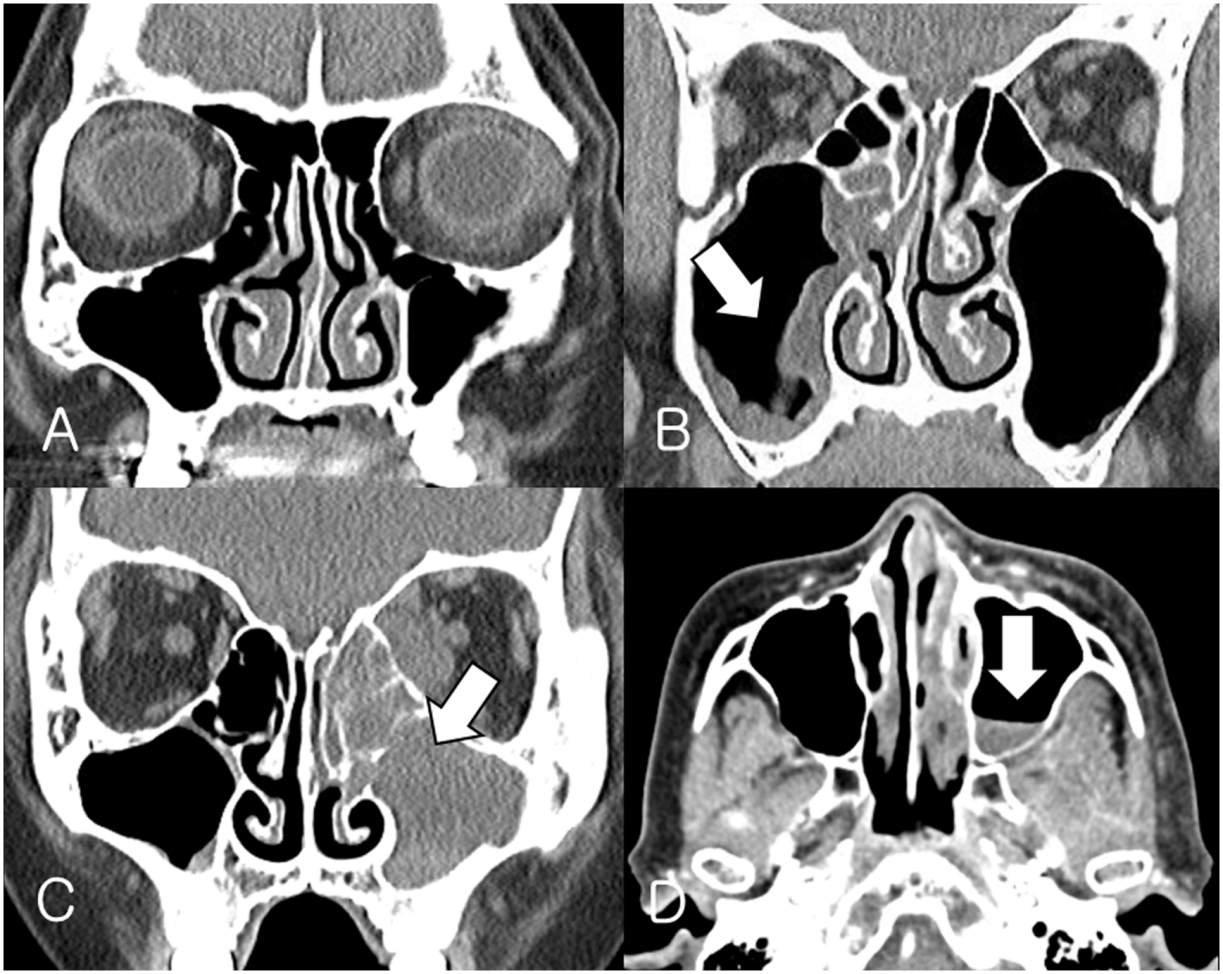
Computed tomography findings of rhino-orbito-cerebral mucormycosis and bacterial orbital cellulitis. A. Clear maxillary and ethmoid sinuses; B. mucosal thickening in the right maxillary sinus; C. full opacification in the left maxillary and ethmoid sinuses; and D. air-fluid level in the left maxillary sinus.

Orbital CT scans were performed in all patients during their first clinical visit, and we checked the presence of a clear paranasal sinus, the air-fluid level, thickening of the sinus mucosa, and full opacification. Sinus mucosal thickening was defined as positive if the mucosa of the ethmoid or maxillary sinus was thickened > 2 mm. Full opacification was defined as opacification within the entire area of the ethmoid or maxillary sinus without air shading. The air-fluid level was defined as the level observed in the ethmoid or maxillary sinus (Fig 1).

### Statistical analysis

SPSS statistical package ver. 18.0 (SPSS Inc., Chicago, IL, USA) was used for the statistical analysis. Various clinical factors were compared between the groups using the Mann—Whitney *U*-test and Fisher’s exact test. Fisher’s exact test and the chi-square test were used to compare clinical and CT scan findings. A *p* < 0.05 was considered significant.

## Results

Fourteen of the 34 patients presented with ROCM and 20 had BOC. The mean ages of the patients with ROCM and BOC were 63.2 ± 10.9 and 47.1 ± 22.9 years, respectively (*p* = 0.059). ROCM was observed in single eyes of 13 patients and in both eyes of one patient, whereas BOC was detected in single eyes of all 20 patients. Ten (71.4%) of 14 patients with ROCM had diabetes mellitus, and their comorbidity rate was significantly higher than that of patients with BOC (n = 1, 5.0%) (*p* < 0.001). The incidence of hypertension was significantly different between the groups (57.1% vs. 20.0%, *p* = 0.036). Other diseases in patients with ROCM included myelodysplastic syndrome (n = 3) and Crohn’s disease (n = 1). The patients with BOC had no other diseases, except for pancreatic cancer in one patient (Table 1).

**Table 1:** Patient demographics.

	ROCM	BOC	*p*-value
No. of patients	14	20	
Age (years)	63.2 ± 10.9	47.1 ± 22.9	0.059*
Sex (M/F)	7/7	10/10	1.000^†^
Diabetes mellitus (n, %)	10 (71.4%)	1 (5.0%)	<0.001^‡^
Hypertension (n, %)	8 (57.1%)	4 (20.0%)	0.036^‡^
Others (n, %)	4 (28.6%)	1 (5.0%)	0.135^‡^
MDS (n, %)	3 (21.4%)	0 (0.0%)	0.061^‡^
Crohn’s disease (n, %)	1 (7.1%)	0 (0.0%)	0.412^‡^
Pancreatic cancer (n, %)	0 (0.0%)	1 (5.0%)	1.000^‡^

Values are presented as mean ± standard deviation.

* Mann—Whitney *U*-test,

^†^ Chi-square test,

^‡^ Fisher’s exact test

Abbreviations: ROCM, rhino-orbito-cerebral mucormycosis; BOC, bacterial orbital cellulitis; MDS, myelodysplastic syndrome

Four patients with ROCM had no medical record of conjunctival injection or chemosis, whereas two patients with BOC had no medical record of ptosis or EOM limitation. Thus, comparisons were only made in patients whose medical records were contained these signs. Eyelid swelling was more common in the BOC group (n = 18, 90.0%) than in the ROCM group (n = 5, 35.7%) (*p* = 0.002). An EOM limitation was observed in all patients with ROCM (n = 14, 100.0%) and in 12 patients with BOC (66.7%) (*p* = 0.024). Differences with respect to other symptoms, such as ptosis without swelling (*p* = 0.178), conjunctival injection (*p* = 0.255), and chemosis (*p* = 0.384), were not significant between the groups (Table 2).

**Table 2:** Symptoms of rhino-orbito-cerebral mucormycosis and bacterial orbital cellulitis.

	ROCM	BOC	*p*-value
Eyelid swelling (n, %)	5/14 (35.7%)	18/20 (90.0%)	0.002*
Ptosis (n, %)	8/14 (57.1%)	6/18 (33.3%)	0.178^†^
EOM limitation (n, %)	14/14 (100.0%)	12/18 (66.7%)	0.024*
Injection (n, %)	4/10 (40.0%)	13/20 (65.0%)	0.255*
Chemosis (n, %)	6/10 (60.0%)	16/20 (80.0%)	0.384*

* Fisher’s exact test,

^†^ Chi-square test

Four patients with mucormycosis had no medical record of conjunctival injection or chemosis, and two patients with cellulitis had no medical record of ptosis or EOM limitation.

Abbreviations: ROCM, rhino-orbito-cerebral mucormycosis; BOC, bacterial orbital cellulitis; EOM, extraocular muscle

Abnormal paranasal sinus findings were detected on the CT scans of all 14 patients with ROCM (100.0%), whereas 12 patients with BOC (60.0%) had abnormal paranasal sinus findings (*p* = 0.011). Thickening of the sinus mucosa was noted in 13 patients with ROCM (92.9%), which was higher than that in the BOC group (n = 9, 45.0%) (*p* = 0.009). No significant differences in full opacification (*p* = 0.251) or air-fluid level (*p* = 0.412) were detected between the groups (Table 3).

**Table 3:** Computed tomography findings of rhino-orbito-cerebral mucormycosis and bacterial orbital cellulitis.

	ROCM (n = 14)	BOC (n = 20)	*p*-value*
Clear (n, %)	0 (0.0%)	8 (40.0%)	0.011
Sinus mucosal thickening (n, %)	13 (92.9%)	9 (45.0%)	0.009
Full opacification (n, %)	0 (0.0%)	3 (15.0%)	0.251
Air-fluid level (n, %)	1 (7.1%)	0 (0.0%)	0.412

* Fisher’s exact test

Abbreviations: ROCM, rhino-orbito-cerebral mucormycosis; BOC, bacterial orbital cellulitis

## Discussion

Mucorales species are ubiquitous and have characteristically wide branching hyphae lacking septa. Mucormycosis can invade various sites in the body and is divided into six subtypes depending on the site involved: (1) rhino-orbito-cerebral, (2) pulmonary, (3) cutaneous, (4) gastrointestinal, (5) disseminated, and (6) uncommon rare forms, such as endocarditis, osteomyelitis, peritonitis, and renal infection.[4, 8] ROCM is the most common among these subtypes.[9]

The prevalence of BOC (3.51 cases/100,000)[10] is much higher than that of ROCM (1.7 cases/1,000,000)[11]. As early signs and symptoms of ROCM are similar to those of BOC, it is quite difficult for clinicians to identify ROCM in a patient with periocular inflammation. However, the rapid progression of ROCM can result in fatal consequences in the absence of aggressive medical intervention at the early stage of the disease. Thus, an early diagnosis is crucial.

In general, ROCM causes infection in immunocompromised patients, and uncontrolled diabetes mellitus is one of the most important risk factors for ROCM.[4] The higher incidence of diabetes mellitus found in the current study for ROCM cases relative to BOC cases coincides with previously reported outcomes. Unlike in previous reports, we found morbidity due to hypertension to be significantly higher in the ROCM group, but no differences were detected with respect to other disease that would result in an immunocompromised state, suggesting that above differences might be an error due to the small sample size of this study.

BOC is an inflammation of the orbital tissues behind the orbital septum, such as the EOM, orbital fat, or the optic nerve. This disease is commonly caused by previous sinusitis. Other causes include periocular trauma, previous surgery, dacryocystitis, and periodontal infection.[12–14] Common signs and symptoms of BOC include proptosis, chemosis, hyperemia, diplopia, and pain during eye movement. If the infection spreads into the cavernous sinus and the cranial cavity from the orbit, complications, such as visual loss, cavernous sinus thrombosis, and meningitis, may occur. Some studies have reported BOC-related visual loss rates of 6.0–23.6% of admitted patients, [14, 15] but the incidence of such severe complications decreases significantly with adequate antibiotic therapy.[16]

Most cases of ROCM originate as ethmoid sinusitis, with the disease extending rapidly through the lamina papyracea into the orbit where the infection can spread to the EOM, the optic nerve, and other orbital tissues. Kulkarni et al.[17] reported that the ethmoid sinus was the first site infected by ROCM in a cadaveric study, and that the infection had spread through the lamina papyracea into the orbit at an earlier period. Subsequently, III, IV, V1, V2, and VI cranial nerve palsy may occur by invasion of the cavernous sinus, which drains from the ethmoidal and orbital veins. Thus, early involvement of ROCM in the orbit and cavernous sinus may rapidly lead to periocular pain, diplopia, ptosis, and EOM limitation.[6] In this study, we found that limited eye movement was more common in patients with ROCM than in those with BOC at the time of initial diagnosis, whereas eyelid swelling was less common in patients with ROCM than in those with BOC. These symptoms may be attributed to the fact that early invasion of ROCM into the orbit or cavernous sinus primarily causes neurological syndromes.

**Fig 2:**
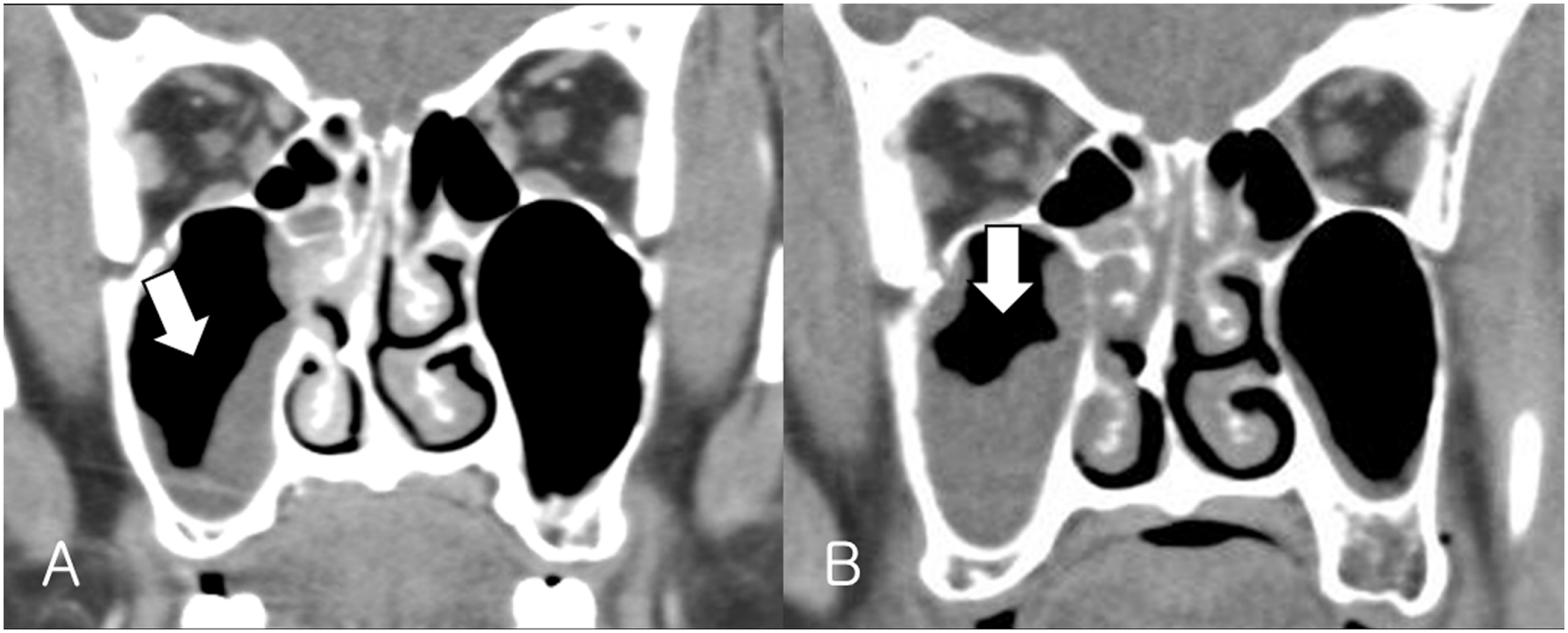
Computed tomography (CT) scans of a patient with rhino-orbito-cerebral mucormycosis and mucosal thickening. A. Initial CT scan of a patient who presented with complaints of eyelid swelling and orbital pain. Coronal view shows mucosal thickening in the right maxillary sinus and opacification in some of the ethmoid air cells; B. Follow-up CT scan 5 days after the first visit. Mucosal thickening in the right maxillary sinus had progressed.

CT scans of patients with early-stage ROCM can detect thickening of the sinus mucosa, full opacification, and air-fluid level. If the infection progresses, the periorbital tissues and bone margins may be destroyed.[18–21] Sinus mucosal thickening is a relatively common finding, as 481 of 982 normal subjects who underwent magnetic resonance imaging showed mucosal thickening of the paranasal sinuses.[22] Our results show that mucosal thickening of the paranasal sinuses occurred in 45% of patients with BOC (9/20 patients) and 13 of 14 patients with ROCM (92.9%). It is unknown why thickening of the paranasal sinuses was more common in patients with ROCM but as mucosal thickening is frequently detected at an early stage and ROCM progresses faster than BOC, patients typically visit the clinic when their ocular symptoms due to ROCM in the orbit occur before the emergence of paranasal sinus opacification. Our hypothesis is supported by the finding that sinus mucosal thickening progressed rapidly within several days when we compared CT scans of a patient with ROCM taken on the first clinic visit with those taken on a visit on day 5 (Fig 2). Thus, if a CT scan reveals mucosal thickening of the paranasal sinuses in a patient showing BOC-like symptoms, clinicians should suspect ROCM.

Black eschar is often a characteristic finding of ROCM and can help with the clinical diagnosis.[4, 6, 17] However, ROCM cannot be ruled out in the absence of black eschar because it tends to be identified during the progression of ROCM.[7] In this study, 12 of 14 patients with ROCM underwent nasal examinations at their first clinic visit, but none of them had black eschar.

Our study had some limitations including a retrospective design and a small sample size. We did not performed fungal and bacterial culture. Therefore, we did not found fungal and bacterial species. However, we confirmed ROCM by characteristic hyphae in histopathologic findings, and BOC by clinical and radiological findings and clinical course. Additionally, interobserver agreement may have been low due to the multicenter design. Comparisons of clinical manifestations between the two groups were only available for eyelid swelling, ptosis, EOM limitation, conjunctival injection, and chemosis, and did not address decreased vision, pain, and proptosis. Additional study will be needed to bolster such comparisons in a larger population.

In conclusion, if an immunocompromised patient or one with uncontrolled diabetes presents with BOC-like symptoms, such as an EOM limitation without eyelid swelling, and mucosal thickening is revealed in the paranasal sinuses on a CT scan, a biopsy should be promptly performed to ensure an early diagnosis of ROCM and immediate treatment.

## Supporting Information

S1 File Specific dataset for all individuals. (XLSX)
